# A Rare Case of Akathisia After Removal of Subdural Hematoma During Treatment for Malignant Pleural Mesothelioma: Akathisia Induced by Brain Injury or Immune Checkpoint Inhibition

**DOI:** 10.1155/crom/8854694

**Published:** 2025-12-05

**Authors:** Tomomi Wada, Yukihiro Yano, Emi Miyamoto

**Affiliations:** ^1^ Department of Supportive and Palliative Medicine, Department of Psychiatry, National Hospital Organization Osaka Toneyama Medical Center, Toyonaka, Osaka, Japan; ^2^ Department of Thoracic Oncology, National Hospital Organization Osaka Toneyama Medical Center, Toyonaka, Osaka, Japan; ^3^ Certified Nurse of Palliative Care, National Hospital Organization Osaka Toneyama Medical Center, Toyonaka, Osaka, Japan

## Abstract

Akathisia is a movement disorder primarily caused by antipsychotic medications. Although extremely rare, cases caused by immune checkpoint inhibitors or traumatic brain injury have also been reported. We report on the case of an 82‐year‐old patient with malignant pleural mesothelioma who developed akathisia following subdural hematoma removal and achieved successful symptom control. Three months after receiving nivolumab and ipilimumab, the patient developed incomplete paralysis of the right side of the body. Examination and medical history revealed that a subdural hematoma was the cause of the movement abnormality. Following hematoma removal, the patient became unable to sit still. We suspected the condition as akathisia secondary to traumatic brain injury. Anticholinergic medication successfully controlled the symptoms, allowing treatment for mesothelioma to resume. Neurological immune‐related adverse events associated with immune checkpoint inhibitors are often difficult to resolve completely and can lead to discontinuation of cancer treatment. We hope this case report underscores the importance of carefully evaluating the pathophysiology of neurological abnormalities arising during cancer treatment.

## 1. Introduction

Akathisia is a movement disorder characterized by an intense sensation of inner restlessness and a strong urge to move. Although the pathophysiology is not fully understood, it is considered part of extrapyramidal dysfunction, with involvement of imbalances in dopamine and norepinephrine in the basal ganglia. Since the advent of antipsychotic medications, it is commonly observed as a side effect of these drugs. In cancer patients, there is widespread awareness of akathisia caused by antipsychotic drugs used for antiemetic or delirium treatment. Akathisia also occurs in association with Parkinson’s disease or encephalitis [1].

Nivolumab, the anti‐PD‐1 antibody, and ipilimumab, the cytotoxic T‐lymphocyte‐associated antigen 4 antibody, are immune checkpoint inhibitors (ICIs) approved for the treatment of malignant mesothelioma. Although ICI therapy causes a variety of immune‐related adverse events (irAEs) including neurological symptoms [2], the association between these drugs and akathisia is unclear. We report on a case of akathisia after subdural hematoma drainage 3 months after the last administration of nivolumab and ipilimumab.

## 2. Case

An 82‐year‐old man diagnosed with malignant pleural mesothelioma underwent first‐line treatment with nivolumab and ipilimumab. Disease progressed on CT evaluation after two courses, and second‐line therapy was scheduled 3 months after the last dose of nivolumab and ipilimumab. During the first‐line treatment, the patient’s mental status was stable. On the day of admission for the second‐line treatment, he exhibited right upper and lower limb motor dysfunction and incontinence, which had not been observed previously. MRI of the brain revealed a subacute to acute subdural hematoma (Figure 1a). On the same day, he was transferred to another hospital for hematoma removal and underwent bilateral cranial drainage surgery. At the transferring hospital, he had a consciousness disorder with a Glasgow Coma Scale (GCS) of 11 (E4V3M4) immediately before surgery and was improved to a GCS of 14 (E4V4M6) level after surgery. However, he was in a severe restless state, and it was explained that he might have delirium or dementia. He was discharged home 9 days after drainage despite a severe restless condition. He walked around every 2–3 min and was restless and insomniac at home; then, he was readmitted to our hospital 9 days after the discharge. Blood biochemistry tests revealed no abnormalities such as electrolyte imbalances that could cause impaired consciousness. Despite treatment with trazodone, lemborexant, ramelteon, and Yokukansan which is the traditional Japanese medicine, he exhibited severe anxiety, agitation, and insomnia, leading to a request for intervention by the palliative care team. On the day of the team intervention, he exhibited impaired orientation, attention deficits, and narrowed consciousness, leading to the diagnosis of mixed delirium. Although risperidone was initiated, agitation accompanied by hallucinations persisted at night, and wandering within the ward continued day and night. Increasing the dose of risperidone and switching to perospirone did not improve insomnia, agitation, and wandering. Moreover, sialorrhea, gait disturbance, and muscle rigidity in the upper limbs appeared, considering the drug‐induced parkinsonism from antipsychotic medications. When perospirone was discontinued and replaced with tiapride, the patient was able to sleep for 30 min to 1 h with nighttime delirium and wandering throughout the day. It took 2 weeks for the nocturnal hallucinations to disappear. The patient’s symptoms were repeatedly interviewed during daytime hours when he was in a better state of consciousness, even before he became completely alert. A detailed interview revealed that the patient felt an internal sense of restlessness that led to pacing, stating, “When I try to lie down in bed, I feel like I shouldn’t sleep,” and “It’s not my legs, but my mind (brain) that feels restless.” Although the patient had lost memories of the period before and after surgery, interviewing with the family revealed that the patient had fallen several times at home and had stated, “I have an obsessive fear of sleeping,” 4–5 days before admission to our hospital prior to drainage. Given the presence of restlessness and wandering prior to antipsychotic medication use, we considered the possibility of akathisia caused by brain injury. We reassessed a brain MRI and initiated low‐dose anticholinergic drug biperiden. The MRI exhibited no signs of encephalitis, brain metastasis, or recurrence of blood accumulation (Figure 1b). As biperiden was gradually increased, sleep duration gradually prolonged, and internal agitation improved. Clonazepam was tried in small doses to ensure adequate sleep and treat akathisia. However, it was discontinued due to nocturnal disorientation. An oversight in prescribing biperiden for 2 days caused increased salivation; then, it improved upon resumption. Two months after cranial drainage, treatment for mesothelioma with pemetrexed was resumed with continued biperiden. Although internal restlessness appeared two to three times a day, a short walk eliminated the need for constant move, and the patient was able to sleep coherently for about 5 h at night.

Figure 1(a) The right subdural space shows findings suggestive of an acute to subacute hematoma with mixed signal intensity on T1‐weighted images and high‐signal intensity on T2‐weighted images. A high‐signal retention with a septum suggests a relatively old hematoma on the left. Blurring of the sulci and ventricular deformation in the left→right cerebral hemisphere suggests compression of the brain. (b) An MRI performed 34 days later showed relatively old blood remaining in both subdural spaces, but the compression of the brain parenchyma had resolved. No meningeal enhancement was observed with gadolinium contrast. Abnormal findings suggestive of encephalitis were not also noted.

**Figure:**
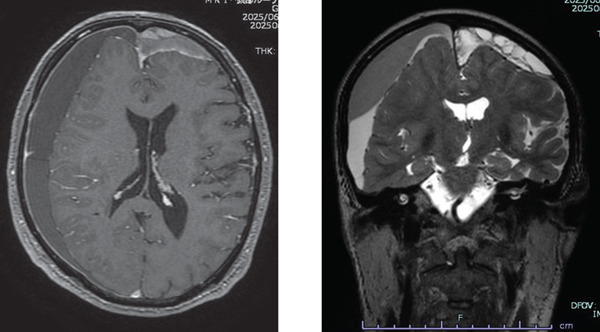
(a)

**Figure:**
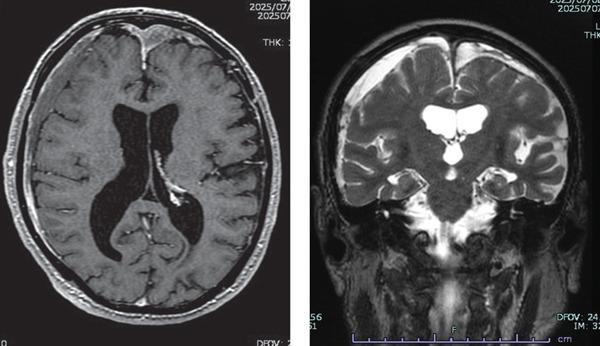
(b)

## 3. Discussion

We describe a case of akathisia developed after subdural hematoma drainage surgery, leading to a delay in mesothelioma treatment. Because akathisia developed during a period when no antipsychotics or other dopamine D2 receptor antagonists were being used, we considered brain damage that caused a subdural hematoma to be the cause of akathisia. According to family interviews, there may have been an inability to remain still for several days before the hematoma was removed. This suggests that upper and lower limb motor disorders and consciousness disorders before drainage might mask the akathisia symptoms. Additionally, persistence of postoperative delirium hindered to identify akathisia as the cause of agitation, and antipsychotic drugs used to treat delirium may have exacerbated akathisia symptoms. Mild traumatic brain injury is considered the risk of developing parkinsonism [3], whereas very rare cases have been reported regarding akathisia [4–6]. It is important to bear in mind that akathisia may be a contributing factor to agitation after brain injury without the prescription of antipsychotics despite its rarity. To identify akathisia under delirium, it was useful to repeatedly ask about the subjective component [7] during relatively good periods of consciousness. Another possibility regarding the pathology of akathisia is neurotoxicity caused by ICI. Because neurological adverse events associated with ICI often occur several months after the first dose, we cannot rule out an association between akathisia and ICI therapy in our case. The irAEs associated with the combination of ipilimumab and nivolumab are considered relatively rare and may be underreported due to ambiguous symptoms and difficulties in definitive diagnosis [2]. To date, two case reports have speculated the possibility of nivolumab‐induced akathisia in which both cases died without effective treatment [8, 9]. In our case, akathisia was controlled, and then, interrupted treatment for mesothelioma was resumed. Because we inferred from the clinical course that the akathisia was caused by brain injury rather than ICI, we preceded the usual symptomatic treatment for akathisia. If symptoms suspicious for ICI‐induced encephalitis remained after hematoma removal, therapeutic agents for irAE such as corticosteroids and intravenous immunoglobulin should also be considered [10, 11]. In cancer patients, autoimmune encephalitis should also be excluded as a cause of acute consciousness disturbance. In our case, we did not perform detailed investigations such as cerebrospinal fluid analysis or electroencephalography (EEG) as the clinical symptoms did not meet the diagnostic criteria of probable autoimmune encephalitis [12]. Nonconvulsive epilepsy must also be considered as a cause of impaired consciousness following head injury. In our case, the patient remained alert after several weeks of postoperative delirium. However, if impaired consciousness recurs, EEG testing would be useful for the exclusion of secondary epilepsy. Pharmacological interventions for akathisia include beta‐blockers, benzodiazepines, anticholinergic drugs, and mirtazapine/trazodone [1]. In this patient, beta‐blockers were avoided due to low blood pressure, and mirtazapine, a 5‐HT2A antagonist, was avoided because trazodone that has the similar receptor profile exacerbated agitation. Because anticholinergic drugs carry the risk of precipitating delirium, the first dose of biperiden was initiated during the day, gradually increased while monitoring the patient’s mental status, urinary retention, constipation, or dry mouth. In this case, as the accidental discontinuation of biperiden caused sialorrhea, a sign of extrapyramidal symptoms, biperiden was continued to ensure a stable general condition for cancer treatment. As some evidence suggests that the pathogenesis of akathisia and other extrapyramidal symptoms is partially distinct [13], it remains to be confirmed whether discontinuation of biperiden will cause a flare‐up of akathisia. As anticholinergic drugs are associated with cognitive impairment and increased risk of falls in the elderly [14], further neurological follow‐up should be necessary to avoid prolonged prescription.

With advances in cancer management and the aging of the population, cancer treatment while managing other related conditions has become commonplace, highlighting the need to reassess the evaluation of various underlying conditions as potential causes of agitation. Neurological adverse events are difficult to determine their cause and may sometimes be irreversible, impairing the patient’s quality of life. Therefore, it is crucial to accumulate knowledge about neurological adverse events occurring during ICI therapy for enhancing the safety of ICI treatment.

## Consent

Written informed consent was obtained from the patient for publishing this case report. Consent forms written in Japanese are disclosed upon request.

## Conflicts of Interest

The authors declare no conflicts of interest.

## Funding

No funding was received for this manuscript.

## Data Availability

All relevant data is included within this article. Further inquiries can be directed to the corresponding author.
